# Correction: Yang et al. Preparation of Biomass Carbon Composites MgO@ZnO@BC and Its Adsorption and Removal of Cu(II) and Pb(II) in Wastewater. *Molecules* 2023, *28*, 6982

**DOI:** 10.3390/molecules29214980

**Published:** 2024-10-22

**Authors:** Jie Yang, Qing Wei, Changan Tian, Dong Li, Hongming Li, Guangchao Qin, Kunhong Hu, Qinyan Zhang

**Affiliations:** 1 School of Energy Materials and Chemical Engineering, Hefei University, Hefei 230601, China; 2 CAS Key Laboratory of Crust-Mantle Materials and Environments, University of Science and Technology of China, Hefei 230026, China; 3 School of Chemistry and Civil Engineering, Shaoguan University, Shaoguan 512005, China; 4 Hefei Rantian Instrument Co., Ltd., Hefei 230031, China

Following publication, concerns were raised regarding the peer-review process related to the publication of this article [1]. Adhering to our standard procedure, the Editorial Board conducted an investigation which determined that, while the peer-review process does comply with MDPI’s Editorial process policy (https://www.mdpi.com/editorial_process), the contribution of one of the four reviewers does not comply with MDPI’s Guidelines for Reviewers (https://www.mdpi.com/reviewers#_bookmark11) nor the expectations of the Editorial Board. As a result, the Editorial Board has decided to remove the contribution of one of the reviewers from the open peer-review record (https://www.mdpi.com/1420-3049/28/19/6982/review_report) and, following discussion with the authors, have removed the citations (references [4,5] in the original publication) recommended by this reviewer from this publication [1]. The Editorial Board has confirmed that the publication of this paper in its modified form is in line with their expectations and MDPI’s Editorial Process policy.

In the original publication [1], the Conflicts of Interest statement of Qinyan Zhang was not included. The updated Conflicts of Interest statement is as follows: “Author Qinyan Zhang was employed by the company Hefei Rantian Instrument Co., Ltd. The remaining authors declare that the research was conducted in the absence of any commercial or financial relationships that could be construed as potential conflicts of interest.”

The authors state that the scientific conclusions are unaffected. The publication and associated webpage have been updated accordingly.
